# Evidence for the Formation of Difluoroacetic Acid in Chlorofluorocarbon-Contaminated Ground Water

**DOI:** 10.3390/molecules24061039

**Published:** 2019-03-15

**Authors:** Ute Dorgerloh, Roland Becker, Melanie Kaiser

**Affiliations:** Bundesanstalt für Materialforschung und -prüfung, BAM, 12489 Berlin, Germany; roland.becker@bam.de (R.B.); melanie.kaiser@campus.tu-berlin.de (M.K.)

**Keywords:** fluoroacetic acid, DFA, TFA, rainwater, ground water, degradation of refrigerants

## Abstract

The concentrations of difluoroacetic acid (DFA) and trifluoroacetic acid (TFA) in rainwater and surface water from Berlin, Germany resembled those reported for similar urban areas, and the TFA/DFA ratio in rainwater of 10:1 was in accordance with the literature. In contrast, nearby ground water historically contaminated with 1,1,2-trichloro-1,2,2-trifluoroethane (R113) displayed a TFA/DFA ratio of 1:3. This observation is discussed versus the inventory of microbial degradation products present in this ground water along with the parent R113 itself. A microbial transformation of chlorotrifluoroethylene (R1113) to DFA so far has not been reported for environmental media, and is suggested based on well-established mammalian metabolic pathways.

## 1. Introduction

Trifluoroacetic acid (TFA) is very stable in the environment with an estimated half-life of several hundreds of years [1,2] and is found worldwide in oceans [3] and water bodies [4], though no accumulation in biota has been observed [2]. A total TFA release of 20,650,000 metric tons by 2050 to the global environment via abiotic breakdown of refrigerants containing a trifluoromethyl moiety [5] has been estimated [2]. In addition, a natural oceanic source for TFA has been suggested on the basis of concentration patterns [3,6].

Little is known about the origin and behavior of environmental difluoroacetic acid (DFA) and its natural source has not been identified to the best of our knowledge. Significant DFA levels were found in the Detroit river [4] and the urban atmosphere [7]. The limited literature on DFA formation suggests reductive defluorination of TFA to DFA and further to monofluoroacetic acid under certain anaerobic conditions [8,9]. The relevance of DFA formation during the pyrolysis of fluoropolymers [10,11] as a source for environmental DFA levels is unclear, and the formation of DFA by the oxidative metabolization of compounds such as the pesticide flupyradifurone containing the difluoromethyl moiety [12] appears negligible compared to currently observed environmental DFA concentrations.

A survey of short-chain fluorocarboxylic acids in urban aqueous environments revealed compartment-depending differing levels and ratios of TFA and DFA. In the following, this observation is outlined and discussed regarding the potential origin of DFA in ground water with historic chlorofluorocarbon (CFC) contamination.

## 2. Results

Table 1 reveals the concentrations of TFA and DFA in rainwater collected with a temporal interval of 24 h. Assuming a depuration effect by the first rainfall after a longer dry period, the TFA concentration decreased in the consecutive rainfall. This TFA concentration range resembled the average concentration levels observed in the USA [13]. The TFA:DFA ratio of about 10:1 in both rainwater samples correlates well with the airborne TFA:DFA ratio reported for urban areas in Ontario, Canada [7]. Both surface waters contain considerable TFA levels, but notably no detectable DFA. Similar observations were made along the Detroit river, where TFA levels always exceeded DFA levels, which were partly not detectable [4].

**Figure 1:**
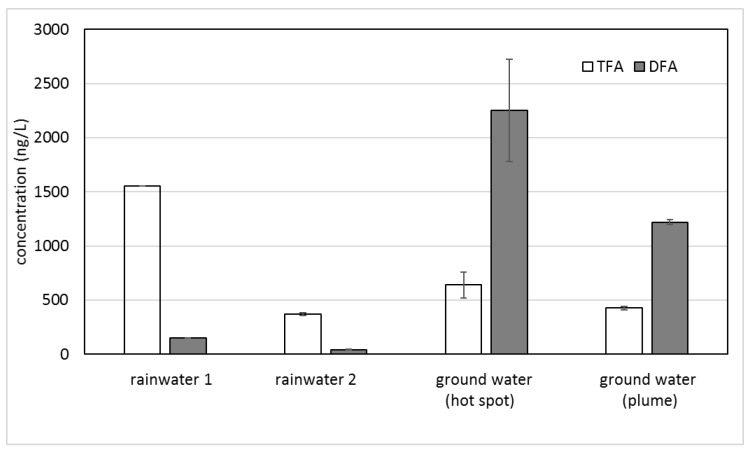
Concentrations of TFA and DFA in rainwater and ground water. Error bars show standard deviation (*n* = 2).

The contaminated ground water contained a TFA level similar to the surface waters but with significantly higher DFA levels (Table 2). The opposite ratios of TFA and DFA in rainwater and ground water are depicted in Figure 1.

**Figure 2:**
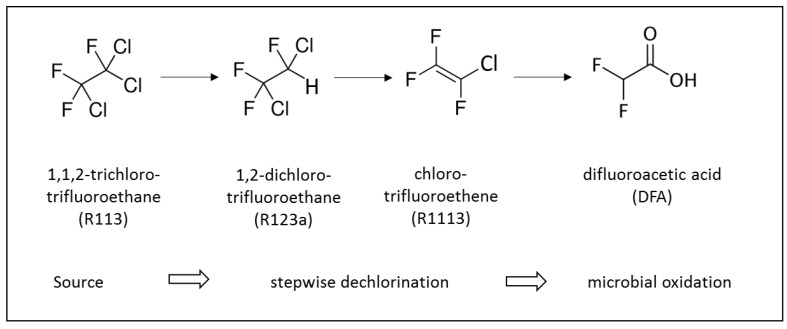
Reductive dechlorination of R113 to R1113 according to Balsiger et al. [15] as observed, and the proposed hydrolysis of R1113 to DFA.

Table 1 and Table 2 and Figure 2 show relatively similar levels of TFA in rainwater, lake water, and ground water. The high TFA concentration in the first rainfall after a long dry period (rainwater 1) and the sewer drain sampling point at the Teltow canal are consistent with a long accumulation period of air contaminants. For the TFA concentration in rainwater 2 collected after a first depuration (rainwater 1), surface water (Havel lake) and the ground water samples displayed a largely similar level of around 500 ng/L (from 370 to 676 ng/L). On the other hand, the DFA concentrations in the ground water samples with a maximum at the contamination hot spot considerably exceeded those of the rainwater. Additionally, the sum of TFA and DFA in the ground water did not correlate with the input from the rain and surface water. Therefore, another source for DFA is likely.

An assessment of this observation may be supported by an inventory of fluorine-containing ground water pollutants at this site (Table 2).

The pollution monitoring at this site supervised by the authors’ institute over two decades revealed 1,1,2-trichloro-1,2,2-trifluoroethane (R113) as sole CFC. Table 2 comprises the average R113 concentrations and the concentrations of 1,2-dichloro-1,2,2-trifluoroethane (R123a) and chlorotrifluoroethene (R1113) as quantified by headspace-GC-MS according to ISO 20595:2018 [14].

Figure 2 depicts the reductive dechlorination sequence of R113 via R123a to R1113 observed under anaerobic conditions in aquifers [15]. The high concentration of DFA in the contaminated ground water suggests a transformation of R1113 to DFA which, to the best of our knowledge, has not yet been reported under environmental conditions.

However, the detoxification pathway of chlorinated 1,2-difluoroethene in rats involves the formation of glutathione-S-conjugates in hepatic cytosol and microsomes [16,17]. In the case of 1,1-dichloro-2,2-difluoroethene, the subsequent hydrolysis to DFA has been reported [18], while in the case of chlorotrifluoroethene (R1113) the respective glutathione-S-conjugate formation was shown but its further fate was not investigated [16]. Nevertheless, DFA is a likely metabolization product of R1113.

Note that glutathione is known to be involved in metabolization reactions of xenobiotics in some microbes as well [19], supporting the idea of the microbial origin of TFA via this pathway.

On the other hand, the abiotic degradation of TFA in water is very slow [20]. The relevance of the reported anaerobic microbial degradation of TFA in laboratory experiments [9,21] for the environmental fate of TFA is not fully clarified [22], and does support reductive defluorination as an alternative source of DFA formation.

The TFA concentration levels at the investigated ground water sites are similar to those of surface water. The local geology displays a low permeability of barrier layers and hydrodynamic flow direction from the canal towards the ground water site. This, and the absence of CFCs containing the CF_3_-group in the ground water as a potential source for TFA, suggest a surface water origin of the TFA in the ground water.

The atmospheric origin of DFA found in the rainwater has to remain an open question. As significant volatilization of TFA and DFA from water bodies can be excluded due to their physicochemical characteristics [23], the possible options are atmospheric oxidation of some difluoromethyl compounds or reductive defluorination of TFA that has not been observed in the atmosphere so far.

## 3. Materials and Methods

### 3.1. Chemicals and Reagents

Methanol (Picograde) was purchased from LGC Promochem (Teddington, UK), ammonium carbonate and concentrated sulfuric acid were from J.T. Baker (Schwerte, Germany), difluoroacetic acid (98%) was supplied by Acros Organics BVBA (Geel, Belgium), and trifluoroacetic acid (99%) was from Alpha Aesar/Thermo Fischer (Karlsruhe, Germany).

### 3.2. Sample Preparation

The water samples were buffered at pH 9 and dried. The fluoroacetic acids were derivatized with methanol to their methyl esters using a modified literature procedure [24].

Each water sample (1 L) was buffered with ammonium carbonate at pH 9 to ensure that TFA was retained completely as acetate during evaporation of the water until the samples were dry. Evaporation was performed in three steps: From 1 L to 50 mL using a recirculating dryer at 70 °C, from 50 mL to 5 mL in a heated nitrogen concentrator at 55 °C (SuperVap 12, FMS, Watertown, MA, USA), and finally after transfer to a 10 mL headspace vial by lyophilization in a freeze drier (LYOVA GT2, SRK Systemtechnik GmbH, Riedstadt, Germany). Each vial containing a dried sample was crimped and cooled down to −28 °C. Then, 800 µL of a mixture of H_2_SO_4_ and methanol (3:1, *v*:*v*) were added with a 1000 µL syringe through the closed septum. The formed fluoroacetic acid methyl esters were volatile and could be determined by gas chromatography from the headspace. For method calibration in a range from 1 to 4000 ng absolute per sample, different amounts from DFA and TFA stock solutions in methanol were added to closed headspace vials through the septa. Then, the derivatization was done as described above. The recovery rate for the drying procedure at 20 and 2000 ng/L was between 70% and 100%, the relative standard deviation (*n* = 3) was between 10% and 17%.

### 3.3. Determination of the Methyl Esters of Difluoro- and Trifluoroacetic Acid by Gas Chromatography–Mass Spectrometry

An HP7890B gas chromatograph coupled with an HP5977A mass selective detector (MSD, Agilent, Santa Clara, CA, USA) and a MPS2XL autosampler (Gerstel, Mülheim, Germany) was used for quantification of TFA and DFA by analyzing their methyl esters. Gas chromatographic separation was achieved using a VF624 standard capillary column (60 m × 320 µm × 1.8 µm) (Agilent). After conditioning of the headspace vial containing the methyl esters (70 °C, 15 min), 1 mL of the headspace volume was injected into the gas chromatograph (split 1:5). The oven program started at 40 °C (held for 10 min) and was ramped to 140 °C (10 °C/min) and further to 240 °C (40 °C/min). The injector, transfer line, and electron source were maintained at 200, 280, and 230 °C, respectively. The MSD (electron impact, 70 eV) was run in the single ion mode (SIM), and the methyl esters of TFA and DFA were quantified using the target ions *m*/*z* 69 and *m*/*z* 51, respectively. The overall detection limit for TFA and DFA was 2 ng per sample. The linear working range was up to 4000 ng per sample. With an initial water volume of 1 L and the matrix effect of real-world water samples, the limit of detection for the full method was 10 ng/L.

### 3.4. Sampling

The rainwater samples were collected in open glass beakers for 1 h (rainwater 1) and for 12 h (rainwater 2), respectively. The unified amounts (sample 1: 1 L, sample 2: 2 L) were immediately processed. The surface water samples were taken headspace-free about 50 cm below the surface in 1 L glass bottles and cooled to 4–6 °C. In the laboratory, the processing started immediately (between 1 and 12 h after sampling). The first surface water sample was from the Teltow canal near a sewer drain close by the rainwater sampling point and the contaminated ground water site. The second sample was from a Havel lake in the periphery of Berlin. The ground water samples were taken by pump sampling (35 m under ground level) from two monitoring wells positioned over the contamination hot spot and the plume (Berlin Oberspree) originating from industrial contamination with R113. The samples were filled headspace-free in 1 L brown glass bottles, closed, and cooled (4 °C) until processed in the laboratory. A separate sample was used for each determination.

## 4. Conclusions

DFA has so far been largely overlooked in the environment, and may be a further relevant product of the microbial degradation of such CFCs that are firstly metabolized to 1,1-difluoroethenes. To the best of our knowledge, this is the first evidence for a relevant formation pathway of DFA in the environment.

## Figures and Tables

**Table 1:** Trifluoroacetic acid (TFA) and difluoroacetic acid (DFA) concentrations in rainfall and surface water (means and relative standard deviations, *n* = 2).

Water Sample	TFA (ng/L)	DFA (ng/L)
Rainwater 1: 1 h of rainfall after a long dry period	1556 *^a^*	151 *^a^*
Rainwater 2: after 12 h of heavy rainfall	370 (3.7%)	43 (4.4%)
Surface water: Teltow canal	1908 (0.9%)	n.d. *^b^*
Surface water: Havel lake	676 (7.1%)	n.d. *^b^*

*^a^* no replicate determination due to the collected volume of just 1 L after 1 h; *^b^* n.d. not detectable (<10 ng/L).

**Table 2:** Concentration of TFA, DFA, and chlorofluorocarbons (CFCs) in the ground water samples, means and relative standard deviations (TFA and DFA: *n* = 2; CFCs: *n* = 6). R113: 1,1,2-trichloro-1,2,2-trifluoroethane; R123a: 1,2-dichloro-1,2,2-trifluoroethane; R1113: chlorotrifluoroethene.

Ground Water Sample	TFA (ng/L)	DFA (ng/L)	R113 (µg/L)	R123a (µg/L)	R1113 (µg/L)
Contamination hot spot	639 (19%)	2249 (21%)	4806 (5%)	27.1 (2%)	68.4 (3%)
Contamination plume	425 (3.3%)	1220 (1.8%)	3315 (3%)	10.1 (4%)	28.2 (3%)
